# Association of trabecular meshwork height with steroid-induced ocular hypertension

**DOI:** 10.1038/s41598-023-36329-4

**Published:** 2023-06-05

**Authors:** Won Jeong Cho, Yitak Kim, Jung Dong Kim, Eun Woo Kim, Hyoung Won Bae, Chan Yun Kim, Wungrak Choi

**Affiliations:** Department of Ophthalmology, Institute of Vision Research, Yonsei University College of Medicine, Seoul, Korea

**Keywords:** Eye diseases, Risk factors

## Abstract

It is important to identify at-risk patients prior to administering steroid injections to prevent avoidable irreversible blindness inducted by steroid-induced ocular hypertension (SIOH). We aimed to investigate the association of SIOH following intravitreal dexamethasone implantation (OZURDEX) using anterior segment optical coherence tomography (AS-OCT). We conducted a retrospective case control study to assess the association between trabecular meshwork and SIOH. A total of 102 eyes that underwent both AS-OCT and intravitreal dexamethasone implant injection were divided into the post-steroid ocular hypertension and normal intraocular pressure groups. Ocular parameters that can contribute to intraocular pressure were measured using AS-OCT. Univariable logistic regression analysis was used to calculate the odds ratio of the SIOH and significant variables were further analyzed using a multivariable model. Trabecular meshwork (TM) height was significantly shorter in the ocular hypertension group (716.13 ± 80.55 μm) than that in the normal intraocular pressure group (784.27 ± 82.33 μm) (p < 0.001). The receiver operating characteristic curve technique analysis showed that the optimal cut-off of ≥ 802.13 μm for TM height specificity was 96.2%, and TM height with < 646.75 μm had a sensitivity of 94.70%. The odds ratio of the association was 0.990 (p = 0.001). TM height was identified as a newly observed association with SIOH. TM height can be assessed using AS-OCT, with acceptable sensitivity and specificity. Caution must be exercised while injecting steroids in patients with short TM height (especially < 646.75 μm) as it may cause SIOH and irreversible blindness.

## Introduction

Dexamethasone (DEX) intravitreal implant injection is a biodegradable, sustained-release corticosteroid implant that provides strong anti-inflammatory effect over approximately 6 months^1,2^. This implant is widely used to treat multiple ophthalmic diseases, including diabetic macular edema (DME)^3^, and serves as an adjunct treatment for patients experiencing macular edema with chronic uveitis or retinal vein occlusions (RVO)^4,5^. Additionally, studies have demonstrated that this implant treatment effectively reduces DME in patients resistant to anti-vascular endothelial growth factor (anti-VEGF) and improves best corrected visual acuity (BCVA)^6^.

Steroid-induced ocular hypertension (SIOH) is a major disadvantage associated with the implants and is defined as elevated intraocular pressure (IOP) of at least 25 mmHg or an increase of at least 10 mmHg from baseline following DEX treatment^7^. Elevated IOP is the only known modifiable risk factor associated with glaucoma, the second leading cause of blindness worldwide as a form of progressive optic neuropathy resulting in visual field defects^8–10^. Thus, SIOH-associated side effects often prevent physicians from readily implementing the DEX implant treatment^11^.

Treatment of patients with SIOH remains controversial. Periodic observation is suggested as withdrawal of steroids in the initial stage may reverse the side effects. However, SIOH was observed in 28.5% of steroid-injected eyes in the MEAD (macular edema: assessment of implantable dexamethasone in diabetes) study^12^, suggesting that if left untreated, the SIOH-related condition might advance to glaucomatous optic neuropathy, called steroid-induced glaucoma (SIG)^13,14^. This highlights the importance of identifying at-risk patients before administering steroid injections and preventing the avoidable irreversible blindness induced by SIOH.

Efforts regarding these vision-threatening complications include the identification of risk factors of primary open angle glaucoma (POAG), a first-degree relative with a history of previous steroid-induced IOP elevation, type 1 diabetes mellitus, connective tissue disease, penetrating keratoplasty, high myopia, and younger age^14–17^.

To our knowledge, SIOH risk factors associated with the differences in ocular anatomy are not established; currently, avoiding DEX treatment is the only identified approach to prevent SIOH. DEX injections may increase IOP by suppressing the phagocytic activity and upregulating the glucocorticoid receptor on the trabecular meshwork (TM), a three-dimensional structure that plays a central role in self-regulation of the aqueous outflow pathway^18–20^. Multiple studies have highlighted a strong association of IOP with TM with respect to increased formation of extracellular matrix and decreased cellularity of the TM observed in glaucomatous eyes^20^. Choi et al. and Chung et al. presented an association between TM height and development of glaucoma^21,22^. Masis et al. reported similar differences in TM height between patients with open and closed angles^23^.

To test the hypothesis that patients with a small TM area are more likely to develop SIOH after intravitreal DEX implant, we aimed to measure the TM height and other ocular parameters, including anterior segment parameters, using anterior segment optical coherence tomography (AS-OCT) and perform statistical tests to determine the optimal cut-off values for the identified risk factors after intravitreal DEX implant.

## Methods

### Ethics statement

The Severance Hospital Institutional Review Board (IRB) approved this retrospective, observational, cross-sectional, double-center study and waived the requirement for informed consent for the review of existing patient records (IRB number, 2021-4372-001). The study protocol adhered to the tenets of the Declaration of Helsinki and complied with the Health Insurance Portability and Accountability Act.

### Patient enrollment

As a case control study, patients who underwent intravitreal DEX (OZURDEX, Allergan, Inc., Irvine, CA, USA) implantation at Yonsei University Health System (Seoul, South Korea) were retrospectively enrolled. The inclusion criteria were eyes that underwent both AS-OCT and received only one DEX implant during a follow-up period of 1 year due to various ophthalmic diseases including DME, RVO, uveitis, and others. Regardless of the type of diagnosis for DEX implants, eyes observed with open angle were included in the study. Glaucomatous eyes (e.g., primary open angle glaucoma (POAG), glaucoma suspect, neovascular glaucoma, secondary glaucoma) except primary angle closure glaucoma (PACG) and primary angle closure (PAC) were also included. Exceptionally, there were eyes with a history of previous primary angle closure suspect (PACS); these eyes all underwent cataract surgery before the study point and were observed with open angle on AS-OCT at the study point. A total of 183 patients (199 eyes) were retrospectively enrolled between March 2013 and February 2021.

The exclusion criteria were as follows: PACG or PAC (i.e., defined as > 180° of the iridotrabecular contact with elevated IOP or presence of peripheral anterior synechiae (PAS)^15,24,25^; 13 eyes), appositional angle closure that eyes with a closed angle (iridotrabecular contact) in either the nasal or temporal side of an eye (25 eyes), history of systemic complication induced by the use of non-topical steroids (3 eyes), baseline IOP of ≥ 23 (12 eyes), history of penetrating trauma (2 eyes), corneal opacities that could disrupt AS-OCT (13 eyes), and poor quality AS-OCT images that hinder the measurement of anterior segment parameters (29 eyes). Finally, 102 eyes (94 patients) were eligible for the final analysis.

### Study design

Data on the following were systematically evaluated from full medical records of 102 eyes (26 eyes with SIOH and 76 eyes without SIOH): age, sex, laterality of eyes, primary diagnosis for DEX implant, underlying systemic diagnosis, glaucoma histories, and complete ocular examination results. For IOP measurement, Goldmann applanation tonometry (GAT) was performed before and after DEX implantation. Gonioscopy exam was done using gonio 3 mirror lens or 4 mirror glass gonio lens. The lens was placed on the eye after anesthesia, and the angle between the iris and cornea was adjusted and examined using a slit-lamp microscope. Abnormalities were assessed, and the exam was repeated in each quadrant of the eye to assess the entire circumference of the anterior chamber angle. The average of three consecutive measurements during the same sitting was considered the final IOP and more readings were taken if the difference between the first two measurements was > 1 mmHg^26^. We subsequently defined and classified IOP elevation as either post-steroid injection IOP ≥ 25 mmHg (8/26 eyes with SIOH or IOP elevation ≥ 10 mmHg over the baseline measurement (18/26 eyes with SIOH)^7^. In each case, we evaluated the IOP at seven points: baseline IOP before injection and IOP at 1 week, 1 month, 2 months, 3 months, 6 months, and 12 months after DEX implant injection. Eyes of enrolled patients were divided into the post-steroid normal IOP group (Group A) and the SIOH group (Group B). Various ocular parameters that can contribute to IOP were measured using AS-OCT.

### TM height and ocular parameter measurements by AS-OCT

All reviewed patients underwent CASIA SS-1000 AS-OCT (Tomey Corporation, Nagoya, Japan)^27^. All procedures were performed at Yonsei University Severance Hospital. To investigate novel risk factors reflecting a patient’s underlying anatomical condition, the AS-OCT images closest in time after the first injection of OZURDEX implant and images with visible TM were selected. Normal-resolution scan mode for the results was used and anterior segment images were obtained using the anterior angle protocol.

The scleral spur (SS), a landmark structure formed from a projection of the sclera, was used as a reference point for both TM height (defined as the distance between SS and Schwalbe’s line [SL]) and automated biometric analyses of the ocular parameters of the anterior chamber angle^28^. To identify SS, we employed a recently described band of extra-canalicular limbal lamina (BELL) method as it is a novel visible landmark on AS-OCT that is adjacent to Schlemm's canal^29^.

**Figure 1:**
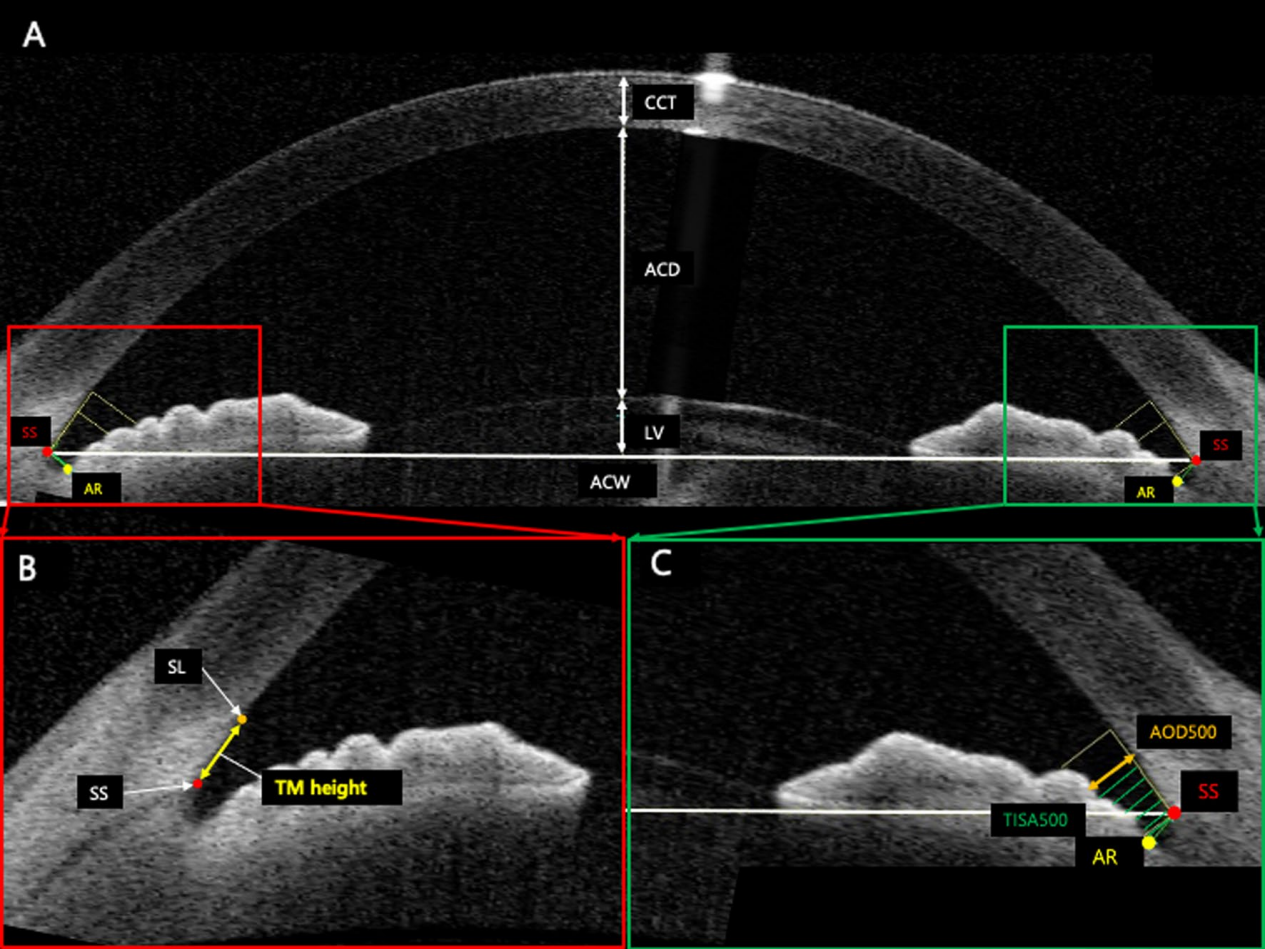
Measurement of TM height and ocular parameters using anterior segment optical coherence tomography (AS-OCT). The overall anterior segment OCT endpoints (**A**). The measurement of the TM height defined as the distance between scleral spur (SS) and Schwalbe’s line (SL) (**B**). Anterior angle parameters of AOD 500 and TISA 500. *SS* scleral spur, *AR* angle recess, *CCT* central cornea thickness, *ACD* anterior chamber depth, *LV* lens vault, *ACW* anterior chamber width, *SL* Schwalbe’s line, *TM* trabecular meshwork, *AOD* angle opening distance, *TISA* trabecular iris space area.

For TM height measurement, we used the standard protocol, the SL method; Crowell et al. and Seager et al. noted that SL is a hypo-reflective structure that reflects the starting point of TM and termination point of corneal endothelium and identifies the inner apex of the U-shaped interface via BELL (Fig. 1)^29^. Therefore, the TM height of enrolled eyes was analyzed with the two important landmarks, SS and SL.

A range of ocular parameters (e.g., angle, anterior chamber, lens, and corneal parameters) measured from both the temporal and nasal sides using AS-OCT were as follows: anterior chamber depth (ACD) (i.e., defined as the distance between the corneal endothelium and the anterior capsule of the lens), anterior chamber width (ACW) (i.e., the distance between the two SS), angle opening distance (AOD) (i.e., the perpendicular distance between the TM), angle recess area (ARA) (i.e., the triangular area bounded by AOD as the base, angle recess as the apex), and the inner corneoscleral wall as the sides of triangle), trabecular iris space area (TISA) (i.e., the trapezoidal area with the boundaries of anterior for the AOD), trabecular iris angle (TIA) (i.e., the angle measured with the apex in the iris recess), lens vault (LV) (i.e., the perpendicular distance between the anterior lens pole and the horizontal line joining the two SS). The central corneal thickness (CCT) of all the patients was also assessed from cross-sectional AS-OCT images by manually computing the width of the cornea. In addition to these parameters, ocular assessments including current and past ophthalmologic diagnosis and baseline IOP measurements were obtained from the evaluated medical records. Finally, IOL Master (Carl Zeiss Meditec AG, Jena, Germany) was used to measure the AXL.

We measured TM height and ocular parameters by determining the 3 and 9o’clock positions as the nasal and temporal positions, respectively. All measurements were performed by two independently trained physicians who were masked to the baseline demographic data, ocular examination results of the patients, and whether the group was A or B. The average value was used for final analysis. To verify the reproducibility of TM height measurements, a subset of 50 eyes was randomly sorted for re-analysis by a trained researcher. Overall, both the intra- and inter-personal agreement was assessed by Bland–Altman comparison analysis with 95% limits of agreement (LoA).

### Statistical analyses

**Figure 2:**
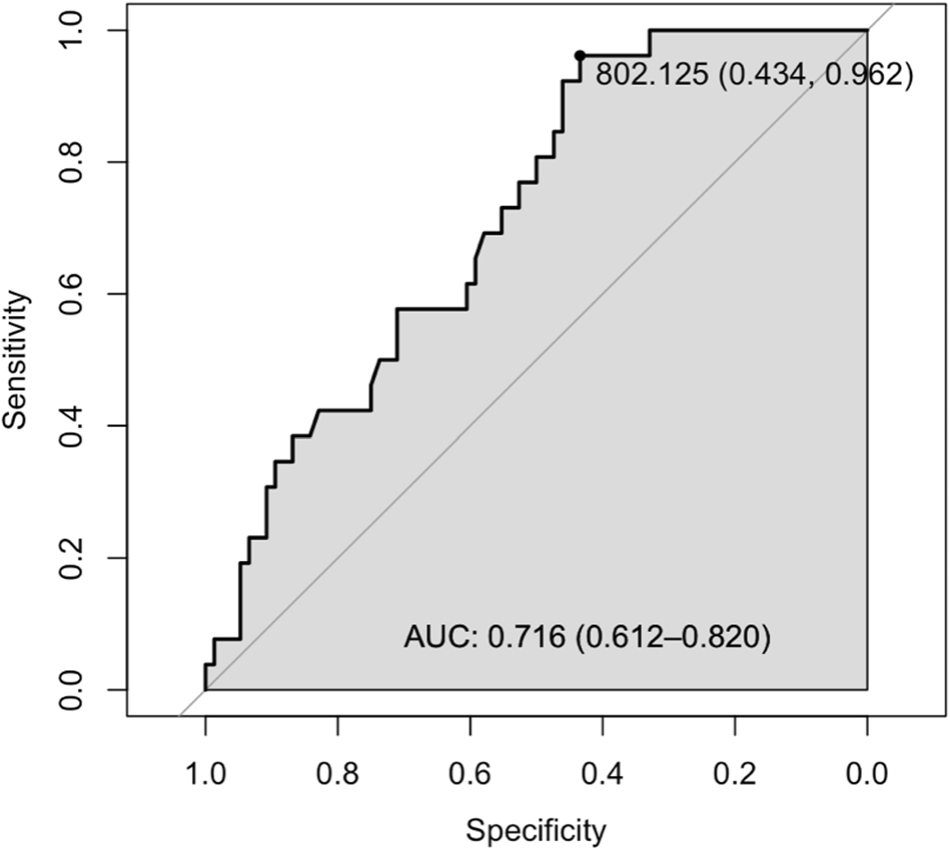
ROC curve of steroid-induced ocular hypertension according to TM height average. *ROC* receiver operating characteristic, *CI* confidence interval, *OR* odds ratio, *AUC* area under the curve.

Data were analyzed using SPSS 22.0 software (SPSS, Chicago, IL), SAS version 9.4 (SAS Institute Inc., Cary, NC), and R package (version 3.6.2, packages; survival; The R Project for Statistical Computing, Vienna, Austria) for data logistic regressions and visualization. Formal statistical tests were performed to compare the baseline clinical characteristics (Table 1) and ocular parameters obtained from AS-OCT (Table 2) of all patients; Group A (non-SIOH) and B (SIOH) were compared in the same manner. We conducted consecutive t-tests and derived p-values using Student's t-test or Welch's t-test with continuous variables. Categorical variables were compared using the chi-squared test if all frequencies in the 2 × 2 contingency table were ≥ e and if any frequency in the 2 × 2 contingency table was < 5, Fisher’s exact test was performed. To investigate the association between clinical factors and SIOH, univariable logistic regression analyses were implemented for variables of ocular parameters (e.g., TM height, ACD, ACW, AOD, ARA, TISA, TIA, and LV) and for variables retrieved from electronic medical records (e.g., baseline IOP, sex, laterality of eyes, AXL, SE, CCT, and systemic diseases) (Table 2). With the odds ratios obtained from the univariable analysis, a stepwise method of the multivariable linear regression analyses to select only variables that were significant and selected significant factors were adjusted for analysis (Table 3). p-values < 0.05 were considered statistically significant. To determine the optimal cut-off value for the average TM height, a receiver operating characteristic (ROC) curve was plotted and the area under the curve (AUC) was then calculated (Fig. 2). We used a univariable logistic regression model to draw the ROC curve setting TM height as the independent variable.

**Table 1:** Study population and demographics.

Baseline Characteristic	All eyes (n = 102 eyes of 94 patients)	Patients without SIOH Group A (n = 76 eyes of 69 patients)	Patients with SIOH^a^ Group B (n = 26 eyes of 25 patients)	p-value
Age (years)	58.43 ± 12.09	59.78 ± 12.11	54.50 ± 11.37	0.054
Sex (M/F) (patients)	43 (45.7%)/51 (54.2%)	32 (46.3%)/37 (53.6%)	11 (44%)/14 (56%)	0.830^†^
Laterality (R/L) (eyes)	63 (61.8%)/39 (38.2%)	48 (63.2%)/28 (36.8%)	16 (61.5%)/10 (38.4%)	0.978^†^
Diagnosis for DEX implants (eyes)				
DME	33 (32.4%)	28 (36.8%)	5 (19.2%)	
RVO	29 (28.4%)	23 (30.3%)	6 (23.1%)	
Uveitis	23 (22.5%)	14 (18.4%)	9 (34.6%)	
Others	17 (16.7%)	11 (10.8%)	6 (23.1%)	
AXL (mm)	23.50 ± 1.20	23.37 ± 1.12	23.92 ± 1.39	0.097
SE (D)	− 0.43 ± 2.82	− 0.13 ± 2.77	− 1.17 ± 2.85	0.153
CCT (μm)	553.18 ± 58.39	556.28 ± 61.52	544.12 ± 48.02	0.362
Baseline IOP (mmHg)	12.95 ± 3.02	12.53 ± 2.93	14.17 ± 3.02	0.016*
Systemic disease				
Hypertension	32 (31.4%)	26 (34.2%)	6 (23.1%)	0.291^†^
Thyroid disease	6 (5.9%)	6 (7.9%)	0 (0.0%)	0.334^§^
Kidney disease	5 (4.9%)	4 (5.3%)	1 (3.8%)	1.000^§^
Cancer	7 (6.9%)	6 (7.9%)	1 (3.8%)	0.675^§^
Diabetes mellitus (type2)	41 (40.2%)	34 (44.7%)	7 (26.9%)	0.110^†^
Cardiovascular problem	16 (15.7%)	14 (18.4%)	2 (7.7%)	0.348^§^
Cerebrovascular accident	7 (6.9%)	5 (6.6%)	2 (7.7%)	1.000^§^
Connective tissue disease	10 (9.8%)	8 (10.5%)	2 (7.7%)	1.000^§^
Glaucoma				
Primary open angle glaucoma	3 (2.9%)	1 (1.3%)	2 (7.7%)	0.159^§^
Glaucoma suspect^b^	12 (11.8%)	9 (11.8%)	3 (11.5%)	1.000^§^
Primary angle closure suspect^c^	5 (4.9%)	4 (5.3%)	1 (3.8%)	1.000^§^
Neovascular glaucoma	2 (2.0%)	1 (1.3%)	1 (3.8%)	0.447^§^
Secondary glaucoma	9 (8.8%)	8 (10.5%)	1 (3.8%)	0.442^§^

Patients who received dexamethasone (DEX) implants 0.7 mg were recruited. *AXL* axial length, *CCT* central corneal thickness, *DEX* dexamethasone, *IOP* intraocular pressure, *SE* spherical equivalent, *SIOH* steroid-induced ocular hypertension, *DME* diabetic macular edema, *RVO* retinal vein occlusion.

^a^The SIOH group was divided as follows: post-injection IOP of ≥ 25 mmHg or IOP elevation of ≥ 10 mmHg over the baseline measurement.

^b^Glaucoma suspect was defined as changes in the optic nerve head, including generalized or focal increases in the optic cup size and increases of more than 0.6 in the cup-disc ratio; the narrowing or notching of the neural rim; optic nerve hemorrhaging; and a cup-disc ratio asymmetry of > 0.2 between the two eyes.

^c^Primary angle closure suspect was defined as eyes with a history of previous primary angle closure suspect that underwent cataract surgery and observed with open angle on AS-OCT at the study point.

Data are presented as mean ± standard deviation or no. (%); *statistical significance; ^†^χ^2^ test; ^§^Fisher’s exact test; otherwise t-test.

**Table 2:** Comparison of ocular parameters between the patients enrolled in Groups A and B.

Ocular parameters	All eyes	Patients without SIOH Group A (n = 76 eyes of 69 patients)	Patients with SIOH Group B (n = 26 eyes of 25 patients)	p-value
TM height (μm)	766.90 ± 86.78	784.27 ± 82.33	716.13 ± 80.55	< 0.001*
ACD (mm)	3.31 ± 0.45	3.30 ± 0.46	3.34 ± 0.42	0.729
ACW (mm)	11.44 ± 0.42	11.44 ± 0.39	11.44 ± 0.51	0.977
AOD 500 (μm)	603.30 ± 225.72	587.95 ± 219.52	648.15 ± 241.77	0.242
ARA 500 (μm)	245.23 ± 109.75	239.23 ± 109.59	262.77 ± 110.44	0.348
TISA 500 (μm)	227.43 ± 95.73	217.96 ± 82.27	255.10 ± 125.10	0.088
TIA 500 (μm)	53.59 ± 14.10	52.96 ± 14.82	55.43 ± 11.80	0.443
LV (mm)	− 0.2987 ± 0.3912	− 0.3054 ± 0.4079	− 0.2790 ± 0.3437	0.772

All values are averaged with the measurements performed at both temporal and nasal sides of eyes; *p < 0.05 from t-test. *ACD* anterior chamber depth, *ACW* anterior chamber width, *AOD* angle opening distance, *ARA* angle recess area, *LV* lens vault, *SIOH* steroid-induced ocular hypertension, *TIA* trabecular iris angle, *TISA* trabecular iris space area, *TM* trabecular meshwork.

**Table 3:** Univariable and multivariable logistic regression of variables that may cause SIOH.

Variables	N	Univariable analysis		Multivariable analysis	
		OR (95% CI)	p-value	OR (95% CI)	p-value
Ocular parameters					
TM height (μm)	102	0.990 (0.984, 0.996)	0.001*	0.989 (0.983, 0.996)	0.001*
ACD (mm)	102	1.195 (0.440, 3.246)	0.726		
ACW (mm)	102	0.984 (0.330, 2.930)	0.976		
AOD 500 (μm)	102	1.001 (0.999, 1.003)	0.244		
ARA 500 (μm)	102	1.002 (0.998, 1.006)	0.347		
TISA 500 (μm)	102	1.004 (0.999, 1.008)	0.099		
TIA 500 (μm)	102	1.013 (0.981, 1.045)	0.440		
LV (mm)	102	1.194 (0.364, 3.913)	0.770		
Baseline characteristics					
Baseline IOP (mmHg)	102	1.212 (1.031, 1.424)	0.020*	1.229 (1.037, 1.456)	0.017*
Sex (male)	102 (45)	0.906 (0.368, 2.228)	0.830		
Laterality (right)	102 (63)	0.987 (0.395, 2.467)	0.978		
AXL (mm)	74	1.454 (0.927, 2.281)	0.103		
SE (D)	72	0.871 (0.720, 1.054)	0.156		
CCT (μm)	102	0.996 (0.987, 1.005)	0.360		
Systemic disease					
HTN	102	0.577 (0.206, 1.613)	0.294		
Kidney disease	102	0.720 (0.077, 6.750)	0.774		
Cancer	102	0.467 (0.054, 4.070)	0.490		
DM (type2)	102	0.455 (0.171, 1.210)	0.114		
Cardiovascular problem	102	0.369 (0.078, 1.747)	0.209		
CVA	102	1.183 (0.215, 6.503)	0.846		
Connective tissue disease	102	0.708 (0.140, 3.572)	0.676		

*Statistical significance.

*SIOH* steroid-induced ocular hypertension, *OR* odds ratio, *CI* confidence interval, *TM* trabecular meshwork, *ACD* anterior chamber depth, *ACW* anterior chamber width, *AOD* angle opening distance, *ARA* angle recess area, *TISA* trabecular iris space area, *TIA* trabecular iris angle, *LV* lens vault, *IOP* intraocular pressure, *AXL* axial length, *SE* spherical equivalent, *CCT* central corneal thickness, *HTN* hypertension, *DM* diabetes mellitus, *CVA* cerebrovascular accident.

## Results

### Baseline characteristics of the study population

Table 1 summarizes the baseline characteristics of a total of 102 eyes of the 94 patients administered with DEX implant (0.7 mg). The mean age and baseline IOP of the patients were 58.43 ± 12.09 years (59.78 ± 12.11 and 54.50 ± 11.37 for Groups A and B, respectively) and 12.95 ± 3.02 mmHg (12.53 ± 2.93 and 14.17 ± 3.02 for Groups A and B, respectively), respectively. Furthermore, the mean AXL and CCT were 23.50 ± 1.20 mm and 553.18 ± 58.39 μm, respectively.

### TM height difference between the patients separated by SIOH

We measured the TM height using the SL and BELL methods to accurately define SS and SL, respectively (Fig. 1)^26,27^. The mean TM height of all eyes was 766.90 ± 88.78 μm; 784.27 ± 82.33 μm and 716.13 ± 80.55 μm for Groups A and B, respectively (p < 0.001; Table 2). The results from Bland–Altman comparison analysis with 95% LoA for both the intra- and inter-personal agreement are as follows: [− 7.05 to 118.71 (55.83)] for inter-personal agreement and [− 18.71 to 15.71 (− 1.50) for intra-personal agreement (p < 0.001; Supplementary Fig. 1a,b, Supplementary Table 1). The mean TM height of eyes after omitting NVG eyes (2 eyes) was 782.86 ± 86.68 μm; 782.86 ± 81.96 μm and 715.63 ± 82.17 μm for Groups A and B, respectively (p < 0.001; Supplementary Table 2). PACS eyes (5 eyes) were also excluded, and the mean TM height was 763.46 ± 86.30 μm; 780.75 ± 81.63 μm and 713.69 ± 81.23 μm for Groups A and B, respectively (p < 0.001; Supplementary Table 2).

### Univariable analyses of variables that may induce SIOH

Variables that showed significant effects on SIOH development (p < 0.05; Table 2) in logistic regression analysis are the baseline IOP (β = 1.212 [1.031, 1.424]) and average TM height (β = 0.990 [0.984, 0.996]) (Table 3). With exclusion of NVG and PACS eyes, the average TM height remains statistically significant (β = 0.990 [0.985, 0.996] and β = 0.990 [0.984, 0.996], respectively) (Supplementary Table 2).

### TM height as a significant factor in multivariable analyses

From both the univariable and multivariable analyses, the TM height and baseline IOP were identified as statistically significant factors (odds ratio 0.989; 95% confidence interval [CI] 0.983, 0.996; p = 0.001; Table 3), the TM height was significantly shorter in eyes with SIOH than those without SIOH following DEX treatment. For baseline IOP, patients with steroid-induced elevation in IOP were more likely to have a higher baseline IOP than those with normal IOP (odds ratio 1.229; 95% CI 1.037, 1.456; p = 0.017; Table 3). The R-squared value was 0.192.

### Determination of the optimal cut-off value for TM height

To determine the optimal cut-off values for the TM height to raise concerns before the administration of DEX implant, we performed a univariable logistic regression model using TM height as the independent variable. An optimal cut-off of ≥ 802.13 μm for TM height average was determined by finding the point of contact of a line with a slope of 1 on the ROC curve, and the specificity associated with this value was 96.2%, respectively, when Youden index was 0.396 (Table 4). On the other hand, TM height with < 646.75 μm was at risk for SIOH with a sensitivity of 94.70% (Table 4). Moreover, the odds ratio of the association was 0.990 (0.984, 0.996, p = 0.001), and the AUC of the model was 0.716 (0.612, 0.820) (Table 5).

**Table 4:** Cut-off points for steroid-induced ocular hypertension by TM height average.

Cut-off point (TM height average)				
Cut-off point		Sensitivity (%)	Specificity (%)	Youden Index
> 567.25	–	98.70	3.80	0.025
> 611.00	–	96.10	7.70	0.037
> 646.75	–	94.70	15.40	0.140
> 675.38	–	90.80	30.80	0.216
> 802.13^†^	Optimal	43.40	96.2	0.396
> 834.63	–	32.90	96.2	0.290
> 861.50	–	19.70	100	0.197

^†^The “optimal” cutoff value was defined by the highest Youden index value (sensitivity + sensitivity − 1) from logistic regression.

**Table 5:** Odds ratios and ROC curve of steroid-induced ocular hypertension according to TM height average.

Outcome	Group A	Group B	OR (95% CI)	p-value	AUC (95% CI)
TM height avg	76	26	0.990 (0.984, 0.996)	0.001	0.716 (0.612, 0.820)

*ROC* receiver operating characteristic, *CI* confidence interval, *OR* odds ratio, *AUC*, area under the curve.

## Discussion

Recent studies show that up to 25% patients who received DEX implantation (OZURDEX) experienced SIOH^7,30,31^; glaucoma or glaucoma-suspicion factors were present in all patients who required surgical intervention^12^. Although baseline characteristics associated with SIOH patients are studied, the data on the association of ocular anatomical structures are scarce. In this study, we have identified TM height as an important anatomical structure that should be measured using AS-OCTs in clinical practice, particularly for patients receiving DEX treatment.

TM is the core structure deeply implicated in the pathogenesis of glaucoma because of its widely accepted roles in IOP control by aqueous outflow pathway regulation^15–17,22^. Although the exact pathophysiology is unclear, evidence indicates that steroids induce molecular and ultrastructural changes by increasing the density of TM cells. Glucocorticoids act on the multiplication of myofibroblasts and accumulation of extracellular matrix along the outer and inner wall of SC, respectively, and modification of the actin cytoskeleton network named cross-linked actin networks in the TM^8,23^. The increased TM cellularity leads to increased stiffness and shrinkage in terms of size, which may result in SIOH^18,19^. Therefore, we considered that the TM height would reflect the size and density of this three-dimensional structure and hypothesized that the prevalence of SIOH is negatively correlated with the TM height.

Our results on the TM height and its importance in SIOH agree with our hypothesis established based on previously reported TM histopathology findings. Hence, after administering intravitreal DEX injection to patients with TM height below the cut-off value, a short-term regular ophthalmic follow-up may be advantageous to detect early increase in IOP and this hypothesis needs to be validated in prospective studies.

Furthermore, many investigators have identified TM via OCT, but only a few studies have determined the measurements of TM^15,23,32^. We adopted the same method as those used in previous studies; we defined TM height as the distance between the SS and SL with SL^33^ and BELL methods^29^ as these anatomical landmarks demarcate TM boundaries to assume the size and volume of the TM.

Among the eyes that underwent both AS-OCT and received DEX implant due to various ophthalmic diseases (e.g., DME, RVO, uveitis, and others), we observed that the mean TM height of all eyes in our study (i.e., the average value of TM height in both nasal and temporal sector of eyes) was 766.90 ± 86.78 μm, which was comparable with a finding of the estimated true mean TM height (752.109 ± 11 μm, of 245 randomly selected eyes) from a study conducted in the same ethnic study population, South Korea^21^. The Bland–Altman analysis in this study showed that the validity and reliability of TM height measurement were in acceptable range, and it is worth noting that both the inter-personal agreement and intra-personal agreement are statistically significant (p < 0.001; Supplementary Table 1).

In addition, a sensitivity analysis was performed after excluding eyes diagnosed as NVG and PACS (2 eyes and 5 eyes, respectively; included in the study due to the absence of PAS or of new vessels in the angle of the anterior chamber at the study point and no recent panretinal photocoagulation (PRP) laser in the last 6 months) to demonstrate the robustness of the results (Supplementary Table 2). The eyes with previous PACS history were observed as open angle at the study point, supposed that cataract surgery that the eyes all underwent helped resolve the angle closure in these eyes. Although TM height is reported to be shorter in patients with previous PACS history, we assume that our result remains its robustness because of the small number of PACS eyes included. Due to the possibility that history of PACS may affect the TM height itself, we aim to investigate the relationship of the two factors in the prospective studies.

This study demonstrates clinical relevancy of using AS-OCT for investigating the differences in ocular anatomy of SIOH patients. This study effectively fills in the gap by presenting the TM height as a surrogate parameter that is readily and noninvasively measurable via AS-OCT for patients needing DEX intervention for a range of ophthalmic diseases including DME, uveitis, or RVO^4–6^. We offered an optimal cut-off of ≥ 802.13 μm for TM height, and the specificity associated with this value was 96.2% (Table 4). With this value, we may encourage clinicians to be comfortable prescribing steroid treatment as patients who have a greater TM height than 802.13 μm would be relatively safe from SIOH. Yet, it is clinically significant to identify patients at risk of SIOH in advance with this novel parameter. This analysis showed that screening patients with TM height below the cut-off point of 646.75 μm may detect patients who may develop SIOH with sensitivity of 94.70% (Table 4). However, extra caution and close monitoring should be exercised by ophthalmologists before implementing steroid injection in patients with TM height under 646.75 μm as there would be a considerable chance of developing SIOH.

Another strength of our study may be the relatively high predictive value of SIOH. The AUC of our model was 0.716 (0.612, 0.820) (Table 5), suggesting a relatively high value for predicting SIOH as a single factor. With this ocular parameter that can serve as a sign for the insidious nature of progressive SIOH, ocular steroid response can be recognized before irreversible damage occurs to the optic nerve.

Our results also suggest that baseline IOP is another candidate factor for OHT induced by DEX. The results are consistent with the notion that POAG is a known individual risk factor associated with post-steroidal IOP elevation^14^, supporting our assumption that patients with higher baseline IOP may have a possibility to develop POAG due to the increased susceptibility to develop SIOH.

This study has some limitations. First, the indirect methodology used in previous studies^21–23,32^ (i.e., use of measurement of TM height to extrapolate and assume the volume of TM) was employed to measure TM. TM is a three-dimensional structure with trapezoidal shape of hyper-reflective tissue; thus, the most accurate measurement would be the one measuring the height, width, and length of TM. Further studies with measurement of TM thickness or width to measure the three-dimensional volume of the TM are suggested for accurate morphometry. Second, although it is widely accepted that AXL had borderline correlation with IOP increase, we could not correlate TM height and AXL in this retrospectively designed study because only 74 of 102 eyes had AXL readings (Table 3). Further prospective analysis with AXL in patients with SIOH should be performed in future studies as high myopia is a known risk factor for SIOH. Third, the R-squared value is in a low level of scale in our multivariate model, which may be because of the absence of other significant variables, including AXL. To overcome this limitation, we aim to improve the SIOH predicting nomogram used in our previous publication^15^ in the near future, incorporating inspection of more risk factors, including TM height. Lastly, only Asian patients were included in this study; thus, a further study across ethnicity is required for generalizability of the findings.

In conclusion, physicians can identify patients at risk of SIOH before administering DEX injection and extra caution should be exercised if the patient has a relatively short TM height, especially if TM height < 646.75 μm. Thus, our findings suggest that a procedure for TM height measurement via AS-OCT would be helpful to predict subsequent SIOH development following DEX implantation.

## Supplementary Information

Supplementary Information.

## Data Availability

The datasets used and/or analyzed during the current study are available from the corresponding author upon reasonable request.
