# Supplementary material for: Association of trabecular meshwork height with steroid-induced ocular hypertension
Source: Sci Rep. 2023 Jun 5;13:9143. doi: 10.1038/s41598-023-36329-4 (PMC10241928; doi:10.1038/s41598-023-36329-4)
Supplement: Supplementary file 1 — Supplementary Information. [file 41598_2023_36329_MOESM1_ESM.docx]

**Supplementary Information**

**Supplementary Figure 1a. Bland-Altman plot for Intra-personal Agreement of Measuring TM height**


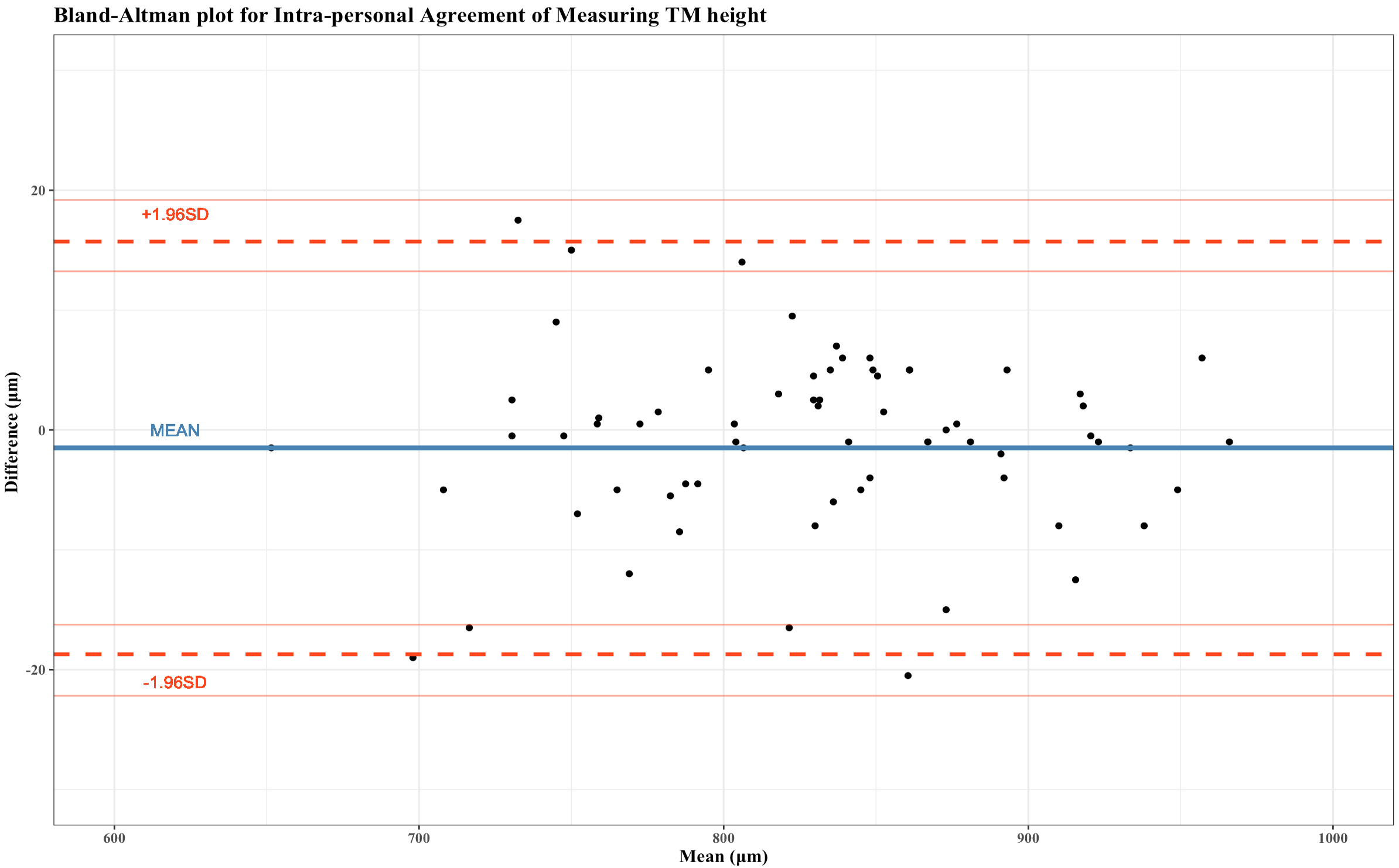


**Supplementary Figure 1b. Bland-Altman plot for Inter-personal Agreement of Measuring TM height**

**
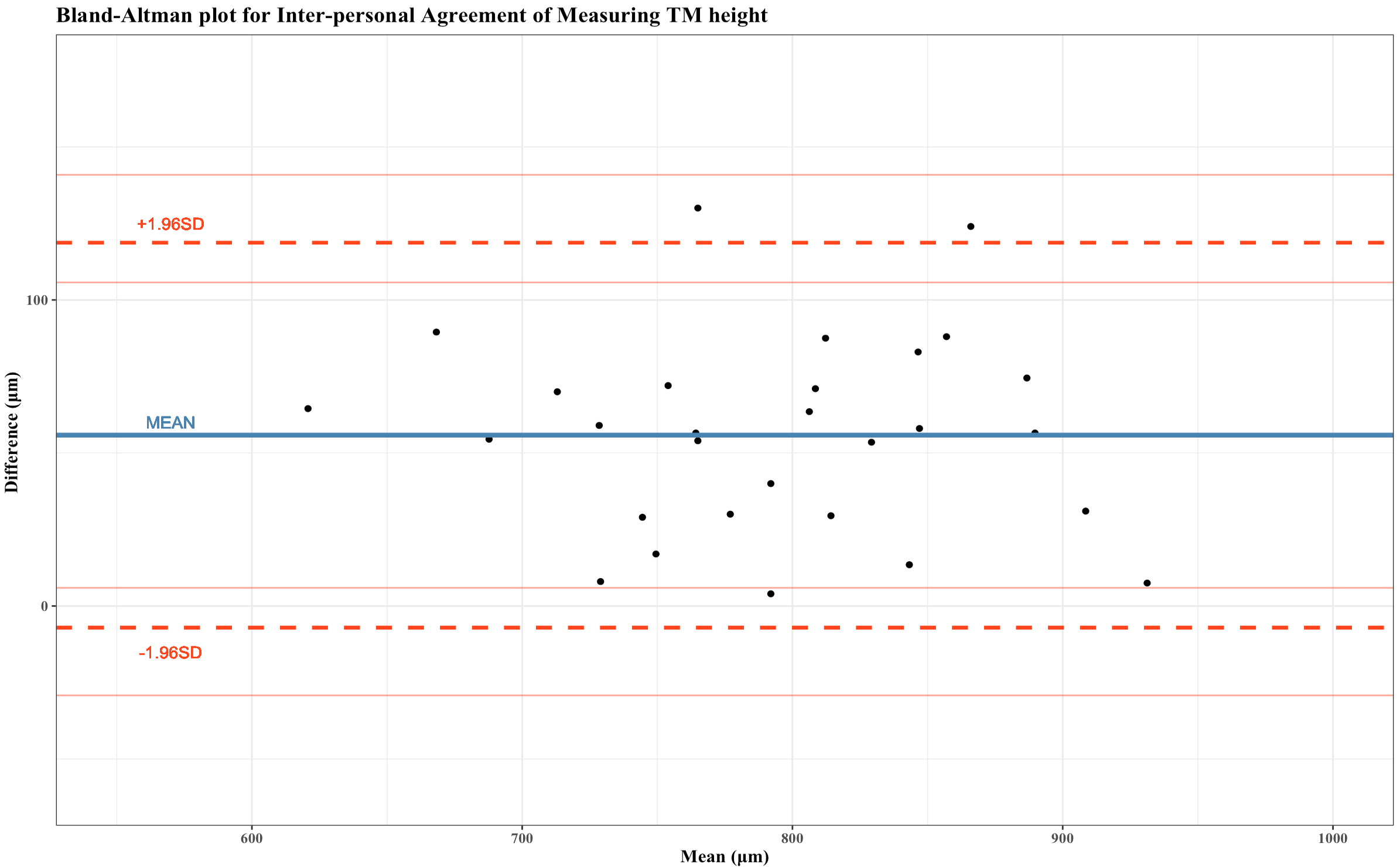
**

| **Type of agreement** | **95%LOA (mean difference)** | ***P*** |
| --- | --- | --- |
| **Inter-personal** | -7.05 to 118.71 (55.83) | <0.001* |
| **Intra-personal** | -18.71 to 15.71 (-1.50) | <0.001* |

**Supplementary Table 1. Interpersonal and intrapersonal agreement expressed through the Bland-Altman comparison analysis**

^*^Statistical significance

LOA, limits of agreement

**Supplementary Table 2. Comparison of TM height before and after excluding eyes with NVG and PACS**

| **t-tests  _____________________________________________** | | | | | | | **Logistic Regression analysis**  **________________________** | | |
| --- | --- | --- | --- | --- | --- | --- | --- | --- | --- |
|  | **N (eyes)** | **All Eyes** | **Group A** | **Group B** | ***P*** | **OR (95% CI)** | | ***P*** |  |
| **All eyes** | 102 | 766.90±86.78 | 784.27±82.33 | 716.13±80.55 | <0.001* | 0.990 (0.984, 0.996) | | 0.001* |  |
| **NVG excluded** | 100 | 782.86±86.68 | 782.86±81.96 | 715.63±82.17 | <0.001* | 0.990 (0.985, 0.996) | | 0.002* |  |
| **PACS^a^ excluded** | 97 | 763.46±86.30 | 780.75±81.63 | 713.69±81.23 | <0.001* | 0.990 (0.984, 0.996) | | 0.002* |  |

^*^Statistical significance

TM, trabecular meshwork; NVG, neovascular glaucoma; PACS, primary angle closure suspect
^a^Primary angle closure suspect was defined as eyes with a history of previous primary angle closure suspect that underwent cataract surgery and observed with open angle on AS-OCT at the study point
